# *Verrucosispora rhizosphaerae* sp. nov., isolated from mangrove rhizosphere soil

**DOI:** 10.1007/s10482-017-0933-4

**Published:** 2017-09-22

**Authors:** Qing-yi Xie, Xiao-dong Bao, Qing-yu Ma, Fan-dong Kong, Man-li Zhou, Bing Yan, You-xing Zhao

**Affiliations:** 1 Institute of Tropical Bioscience and Biotechnology, Chinese Academy of Tropical Agricultural Sciences, Haikou, 571101 People’s Republic of China; 2 Guangxi Key Lab of Mangrove Conservation and Utilization, Guangxi Mangrove Research Center, Beihai, 536000 People’s Republic of China

**Keywords:** *Verrucosispora rhizosphaerae* sp. nov., Polyphasic taxonomy, 16S rRNA

## Abstract

An actinomycete strain, 2603PH03^T^, was isolated from a mangrove rhizosphere soil sample collected in Wenchang, China. Phylogenetic analysis of the 16S rRNA gene sequence of strain 2603PH03^T^ indicated high similarity to *Verrucosispora gifthornensis* DSM 44337^T^ (99.4%), *Verrucosispora andamanensis* (99.4%), *Verrucosispora fiedleri* MG-37^T^ (99.4%) and *Verrucosispora maris* AB18-032^T^ (99.4%). The cell wall was found to contain *meso*-diaminopimelic acid and glycine. The major menaquinones were identified as MK-9(H_4_), MK-9(H_6_) and MK-9(H_8_), with MK-9(H_2_), MK-10(H_2_), MK-9(H_10_) and MK-10(H_6_) as minor components. The characteristic whole cell sugars were found to be xylose and mannose. The phospholipid profile was found to contain phosphatidylethanolamine, diphosphatidylglycerol, phosphatidylinositol mannoside, phosphatidylinositol, phosphatidylserine and an unidentified phospholipid. The DNA G+C content was determined to be 70.1 mol%. The results of physiological and biochemical tests and low DNA-DNA relatedness readily distinguished the isolate from the closely related species. On the basis of these phenotypic and genotypic data, strain 2603PH03^T^ is concluded to represent a novel species of the genus *Verrucosispora*, for which the name *Verrucosispora rhizosphaerae* sp. nov. is proposed. The type strain is 2603PH03^T^ (=CCTCC AA 2016023^T^ = DSM 45673^T^).

**Electronic supplementary material:**

The online version of this article (doi:10.1007/s10482-017-0933-4) contains supplementary material, which is available to authorized users.

## Introduction

The genus *Verrucosispora* was established by Rheims et al. (1998) as a member of the family *Micromonosporaceae*. Members of the genus *Verrucosispora* form a well-developed substrate mycelium, lacking aerial mycelium or sporangia. The major menaquinones are MK-9(H_4_) and MK-9(H_6_), *meso*-diaminopimelic acid is the diagnostic diamino acid of the peptidoglycan. The phenotypic, chemotaxonomic and phylogenetic characteristics of the genus (Goodfellow et al. 2012; Stackebrandt 2012) and genus-specific primers (Xie et al. 2011) allow the genus to be distinguished from other genera classified in the family *Micromonosporaceae*. There is considerable interest in members of the genus *Verrucosispora* as they are a source of novel antibiotics including, for example, abyssomicins (Bister et al. 2004) and Proximicin A (Schneider et al. 2008) from *Verrucosispora maris* (Goodfellow et al. 2012), gifhornenolones A and B from *Verrucosispora gifhornensis* (Shirai et al. 2010), proximicins A-C from *Verrucosispora fiedleri* MG-37 (Fiedler et al. 2008), Thiocoraline A from *Verrucosispora sp.* WMMA107 (Wyche et al. 2011), Butrepyrazinone from *Verrucosispora sp.* K51G (Kyeremeh et al. 2014) and Brevianamide F from *Verrucosispora sp.* MS100047 (Huang et al. 2016).

In the course of investigating mangrove as an actinomycete resource in Hainan Province, China (Hong et al. 2009), strain 2603PH03^T^ was isolated from a mangrove rhizosphere soil sample. We present here a polyphasic taxonomic characterisation of strain 2603PH03^T^.

## Materials and methods

### Isolation and maintenance of isolate

A mangrove rhizosphere soil sample was collected in Wenchang, Hainan, China (GPS: N19°36.506′, E110°47.746′). The soil sample was air dried at room temperature for a week. The dried soil sample (0.1 g) was added to 0.9 mL of sterile water. The resultant 10^−1^ dilution was initially ribolised with a FastPrep-Instrument for 2 s at a speed of 4.0 m/s, and then diluted to 10^−2^ and 10^−3^ (Xie et al. 2011). The soil suspensions were spread on the surfaces of agar plates of PH medium. The medium consisted of l-arabinose (1.0 g), salicin (1.0 g), l-phenylalamine (0.1 g), l-histidine (0.1 g) and 15 g agar, supplemented with base mineral salts (Na_2_HPO_4_, 0.8 g; KH_2_PO_4_, 0.2 g; MgSO_4_·7H_2_O, 0.2 g; CaCl_2_·2H_2_O, 0.2 g; FeCl_3_·6H_2_O, 5.0 mg; Na_2_MoO_4_·2H_2_O, 1 mg), potassium dichromate (30 ml/l), novobiocin (5 mg/l) and nystatin (30 mg/l), in 1.0 L distilled water at pH 7.4. After 4 weeks of aerobic incubation at 28 °C, the isolate, which formed a vivid orange yellow colony, was transferred and purified on yeast extract-malt extract (ISP 2) agar (Shirling and Gottlieb 1966) and maintained as working cultures on ATCC 172 medium (http://www.atcc.org).

The reference strains *V. gifhornensis* DSM 44337^T^, *V. maris* AB18-032^T^ and *V. fiedleri* MG-37^T^ were obtained from Prof Michael Goodfellow (University of Newcastle, UK). Reference strains were cultured under the same conditions as strain 2603PH03^T^ in comparative tests.

### Phenotypic characteristics

Cultural characteristics of strain 2603PH03^T^ and the reference strains were determined following growth on tap-water agar, Czapek’s agar (Raper and Fennell 1965), GYM agar (Ochi 1987), ATCC 172 medium, M 8 agar (Castiglione et al. 2008), modified Bennett agar and ISP 1–7 media for 14–21 days at 28 °C. The *ISCC*-*NBS* colour charts were used to determine the designations of colony colours (Kelly^1964^). The morphological characteristics of strain M4I47^T^ were assessed by scanning electron microscopy (Zeiss, Evo18) of 21-day-old cultures grown on ISP 2 medium. The Gram reaction was performed according to Gregersen (1978) by using KOH for cell lysis. The pH (4–11) and NaCl (0–10%) tolerance for growth were determined on ISP 2 medium for 14–21 days at 28 °C. Allantoin hydrolysis was carried out by the method of Gordon (1967). Tests for the degradation (%, w/v) of adenine, elastin, gelatin, guanine, starch, l-tyrosine, uric acid, xanthine and xylan used modified Bennett’s agar as the basal medium (Tan et al. 2006). Carbon source utilisation was tested by using ISP 9 medium (Shirling and Gottlieb 1966) supplemented with 1% (final concentration) carbon source. The utilisation of amino acids as nitrogen source was tested as described by Williams et al. (1983). The other physiological and biochemical characteristics of strain 2603PH03^T^ and the reference strains were tested by using media and methods described by Williams et al. (1983) and Kämpfer et al. (1991).

### Chemotaxonomy

Biomass for molecular systematic and most of the chemotaxonomic studies was obtained and washed after growing in yeast extract malt extract broth (ISP 2) at 28 °C for 7–14 days on a rotary shaker (220 rpm). Cell wall amino acid and whole cell sugars were analysed as the procedure of Lechevalier and Lechevalier (1980). The *N*-acyl group of the muramic acid in the peptidoglycan was determined by the method of Uchida and Aida (1977). The presence of mycolic acids was determined by the method of Minnikin et al. (1975). Phospholipids in cells were extracted and identified by the method of Minnikin et al. (1984). Fatty acids were extracted by the method of Sasser (1990) and the composition was determined by Sherlock Microbial Identification System (MIDI). The fatty acid methyl esters were identified by using the Microbial Identification software package (Sherlock Version 6.0; MIDI database: ACTIN6). Menaquinones were extracted according to Minnikin et al. (1984) and analysed by an established HPLC procedure (Wang et al. 2011).

### Phylogenetic analyses

Genomic DNA extraction, PCR-mediated amplification of the 16S rRNA gene and sequencing of the PCR products were carried out as described by Nakajima et al. (1999). The 16S rRNA gene sequence of strain 2603PH03^T^ was aligned with multiple sequences obtained from the GenBank/EMBL/DDBJ databases using CLUSTAL-X software (Version 2.1; Larkin et al. 2007). Alignment was manually verified and adjusted prior to the construction of phylogenetic trees. The phylogenetic trees were generated with the neighbor joining (Saitou and Nei 1987), maximum-likelihood (Felsenstein 1981) and maximum-parsimony tree-making algorithms (Kluge and Farris 1969) using MEGA version 6.0 software (Tamura et al. 2013). Phylogenetic distances were calculated with Kimura’s 2-parameter model (Kimura 1980) and the stability of the tree topologies was evaluated by bootstrap analysis (Felsenstein 1985) based on 1000 resamplings. *Salinispora arenicola* CNB-643^T^ was used as an outgroup. The values for the 16S rRNA gene sequence similarities between strains were determined using the EzBioCloud (Yoon et al. 2016).

### DNA relatedness studies

Genomic DNA of strain 2603PH03^T^ was obtained as described by Pospiech and Neumann (1995). The DNA G+C content of strain 2603PH03^T^ was determined by the HPLC method (Mesbah et al. 1989a, b). The level of DNA relatedness between strain 2603PH03^T^ and the related strains were measured on nylon membranes using the method described by Wang et al. (2011).

## Results and discussion

The morphological properties of strain 2603PH03^T^ are consistent with its classification as a member of the genus *Verrucosispora* (Rheims et al. 1998). Strain 2603PH03^T^ was observed to produce well-developed and branched substrate mycelium on ISP 2 medium, with colonies approximately 0.3–0.4 μm in diameter, but no aerial hyphae. Single unevenly warty–surfaced spores are formed on the substrate hypha, with a diameter of approximate 0.6–0.8 μm (Supplementary Fig. S1). Good growth was observed on ISP 1, ISP 2, M 8,ATCC 172, GYM and modified Bennett agar; moderate growth was observed on ISP 5, ISP 6, ISP 7 and tap water agar; poor growth was observed on ISP 3 and ISP 4 media (Supplementary Table S1). The colour of the substrate hyphae was vivid orange yellow to strong orange yellow. No soluble pigment was produced on any of the ISP media tested. Other physiological characteristics are given in the type strain description and Table 1. In addition, the strain is positive for adenine, starch, casein, urea, gelatin, xanthine, elastin, l-tyrosine hydrolysis and nitrate reduction, but negative for allantoin, aesculin, arbutin, guanine, xylan hydrolysis and H_2_S production. d-glucose, l-arabinose, l-fucose, d-fructose, d-mannose, d(+)-melibose, d(+)-arabinose, lactose, d-xylose, maltose, frucose sucrose, melezitose, turanose, d-raffinose, sorbitol, adonitol, glycerol, maltitriose, amygdalin, erythritol, ethanol, arbutin and salicin can be utilised as sole carbon sources but d(+)-galactose, l-rhamnose, d-mannitol, d(+)-trehalose, cellobiose, l-ribose and dulcitol are not. l-alanine, l-arginine, l-histidine, l-phenylalanine, l-serine, l-threonine, l-tyrosine and l-proline are utilised as sole nitrogen source but l-cysteine, l-glycine, l-methionine, l-valine, l-asparagine, and l-glutamic acid are not.

**Table 1:** Differential characteristics of strain 2603PH03^T^ and its close relatives

Characteristics	2603PH03^T^	*V. gifhornensis* DSM 44337^T^	*V. maris* AB18-032^T^	V. fiedleri MG-37^T^
Polar lipids	PE, DPG, PIM, PI, PS, PL	PE, DPG, PIM, PS, PL^a^	PE, DPG, PIM, PS, PG^b^	PE, DPG, PIM, PI, PS, PL^c^
Major menaquinones	MK-9(H_4_), MK-9(H_6_), MK-9(H_8_), (65:18:10)	MK-9(H_4_), MK-9(H_6_), MK-10(H_4_), MK-9(H_2_), (77:7:5:4)^a^	MK9(H_4_), MK-9(H_6_), MK-9(H_2_), (60: 6:1)^b^	MK-9 (H4), MK-9 (H6), MK-10 (H4), (27:10:2)^c^
DNA G+C content (mol%)	70.1	70^a^	70.9^b^	72.0^c^
Biochemical tests				
Aesculin hydrolysis	−	+	−	−
Allantoin hydrolysis	−	+	+	+
Arbutin hydrolysis	−	+	+	−
Casein hydrolysis	+	−	−	−
Elastin hydrolysis	+	−	−	+
Guanine hydrolysis	−	+	+	−
Nitrate reduction	+	−	−	−
Starch hydrolysis	+	+	+	−
Urea hydrolysis	+	−	−	+
Xylan hydrolysis	−	−	−	+
Growth on sole carbon sources at 1%, w/v				
Adonitol	+	−	+	−
Amygdalin	+	+	+	−
l-arabinose	+	+	−	−
d-arabitol	+	−	+	−
Arbutin	+	+	+	−
Dulcitol	−	+	+	−
Erythritol	+	+	+	−
Fructose	+	−	−	−
l-fucose,	+	−	+	+
Glycerol	+	−	+	+
Lactose	+	−	+	+
Maltitriose	+	+	+	−
Mannitol	−	−	+	−
Melezitose	+	−	+	+
Melibiose	+	−	+	+
α-γ-methyl-d-glucoside	−	+	+	−
Raffinose	+	+	−	+
Salicin	+	−	−	+
Sorbitol	+	−	−	−
Trehalose	−	+	+	+
Turanose	+	−	+	+
Growth on sole carbon and nitrogen sources				
l-alanine	+	−	+	+
l-arginine	+	−	+	+
l-asparagine	−	+	+	−
l-cysteine	−	+	+	−
l-glutamic acid	−	+	−	+
l-glycine	−	+	+	+
l-histidine	+	+	+	−
l-methionine	+	+	+	−
l-phenylalanine	+	−	+	+
l-serine	+	+	−	−
l-valine	−	+	+	+
NaCl range(%, w/v)	0–10	0–4	0–5	0–6
pH range	7–10	7–9	7–10	7–10

*Strains 1* 2603PH03^T^; *2 V. gifhornensis* DSM 44337^T^; *3 V. maris* AB18-032^T^; *4* V. fiedleri MG-37^T^; + positive; − negative

*PE* phosphatidylethanolamine; *DPG* diphosphatidylglycerol; *PIM* phosphatidylinositol mannoside; *PS* phosphatidylserine; *PI* phosphatidylinositol; *PG* phosphatidyl glycerol; *PL* unidentified phospholipid

^a^Data from Rheims et al. (1998)

^b^Data from Goodfellow et al. (2013)

^c^Data from Goodfellow et al. (2012); all other phenotypic data were determined in this study

The cell wall of the novel isolate was found to contain *meso*-diaminopimelic acid and glycine. The whole cell sugars were identified as xylose and mannose. The acyl type of the cell wall peptidoglycan was found to be glycolyl. Mycolic acids were not detected. The major menaquinones (>5%) were identified as MK-9(H_4_) (65.0%), MK-9(H_6_) (17.6%) and MK-9(H_8_) (9.6%), with MK-9(H_2_) (2.6%), MK-10(H_2_) (2.4%) and MK-9(H_10_) (2.3%) MK-10(H_6_) (0.5%) as minor components. The characteristic phospholipids were identified as phosphatidylethanolamine (PE), diphosphatidylglycerol (DPG), phosphatidylinositol mannosides (PIMs), phosphatidylinositol (PI), phosphatidylserine (PS) and an unidentified phospholipid (PL), corresponding to phospholipid type PII of Lechevalier et al. (1977) (Supplementary Fig. S2). Significant cellular fatty acids were identified as iso-C_16:0_ (21.4%), iso-C_15:0_ (20.7%), iso-C_18:0_ (14.4%), C_17:1**ω10**c_ (8.8%) C_16:0_ (8.6%), C_17:0_ (5.4%), C_15:0_ (5.1%), anteiso-C_15:0_ (3.9%), anteiso-C_17:0_ (2.6%), C_18:0_ (2.5%), iso-C_14:0_ (1.9%), anteiso-C_16:0_ (1.1%), C_18:1**ω**9c_ (1.1%), iso-C_17:0_ (1.0%)10-methyl-C_16:0_ (1.0%), C_14:0_ (0.4%) and C16:1ω9c (0.3%). The G+C content of the DNA was determined to be 70.1 mol%.

**Fig. 1:**
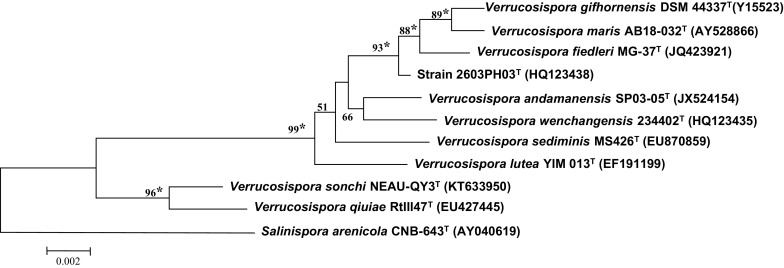
Neighbour-joining phylogenetic tree (Saitou and Nei 1987), based on almost-complete 16S rRNA gene sequences (1407 nt), showing the relationships between strain 2603PH03^T^ and other members of the genus *Verrucosispora. Salinispora arenicola* CNB-643^T^ was used as an outgroup. Numbers at branch points indicate bootstrap percentages (based on 1000 replicates); only values >50% are indicated. *Bar* 0.002 substitutions per nucleotide position. *Asterisks* indicate branches of the tree that were also found maximum-likelihood (Felsenstein 1981) and maximum-parsimony methods (Kluge and Farris)

The almost complete 16S rRNA gene sequence of strain 2603PH03^T^ (1475 nt, GenBank accession number HQ123438) was compared with sequences of representatives of the genus *Verrucosispora.* The 16S rRNA gene sequence similarities of strain 2603PH03^T^ with the type strains of *V. gifthornensis*, *Verrucosispora andamanensis* (Supong et al. 2013), *V. fiedleri* (Goodfellow et al. 2013), *V. maris* (Goodfellow et al. 2012), *Verrucosispora wenchangensis* (Xie et al. 2012), *Verrucosispora sediminis* (Dai et al. 2010)*, Verrucosispora lutea* (Liao et al. 2009) and *Verrucosispora qiuiae* (Xi et al. 2012) were 99.4, 99.4, 99.4, 99.2, 99.1, 99.0 and 99.0%, respectively. Based on 16S rRNA gene sequence analysis, strain 2603PH03^T^ formed a phyletic line on the periphery of the 16S rRNA gene subclade comprised of the type strains of *V. gifhornensis*, *V. fiedleri* and *V. maris*, a relationship which was supported by all of the tree-making algorithms and by a 93% bootstrap value (Fig. 1; Supplementary Fig. S3a and b). Further study showed that the DNA relatedness between strain 2603PH03^T^ and the type strains of *V. gifthornensis*, *V. fiedleri*, *V. maris* were 21.8 ± 2.0, 16.6 ± 4.5, and 18.9 ± 4.0%, respectively, all of which are below the 70% threshold value proposed by Wayne et al. (1987) as the key marker for the identification of a novel prokaryotic species.

The characteristics shown in Table 1 indicated that strain 2603PH03^T^ has some different physiological and biochemical characteristics compared to its closely related phylogenetic neighbours, and can be distinguished on the basis of its inability to degrade allantoin, capacity to grow in fructose and sorbitol as sole carbon source and inability to use l-valine as sole nitrogen source. It is evident from the phenotypic, chemotaxonomic, genotypic and phylogenetic data presented above that strain 2603PH03^T^ can be distinguished from previously described *Verrucosispora* species. Therefore, strain 2603PH03^T^ is concluded to represent a novel species of the genus *Verrucosispora*, for which the name *Verrucosispora rhizosphaerae* sp. nov. is proposed. The Digital Protologue database TaxoNumber (Rosselló-Móra et al. 2017) for strain 2603PH03^T^ is TA00249.

### Description of *Verrucosispora rhizosphaerae* sp. nov.

*Verrucosispora rhizosphaerae* (rhi.zo.sphae’rae. Gr. n. *rhiza*, a root; L. n. *sphaera*, a ball, sphere; N.L. fem. n. *rhizosphaera*, rhizosphere; N.L. gen. n. *rhizosphaerae*, of the rhizosphere, pertaining to the soil from which the type strain was isolated).

Aerobic, Gram-positive, mesophilic actinomycete that forms well-developed and branched substrate hyphae; aerial mycelium and spore vesicles are not formed. Single spores are formed on the substrate hyphae. Grows well on ISP 1, ISP 2, M 8,ATCC 172, GYM and modified Bennett media agar. The substrate hyphae are vivid orange yellow to strong orange yellow. The pH range for growth is 7-10, with an optimum at 7. The maximum NaCl concentration for growth is 10%. Unable to degrade allantoin. Can grow using fructose and sorbitol as sole carbon source. Unable to use l-valine as sole nitrogen source. The cell wall contains *meso*-diaminopimelic acid and glycine. The whole cell sugars are xylose and mannose. The acyl type of the cell wall peptidoglycan is glycolyl. Mycolic acids are not present. The major menaquinones (>5%) are MK-9(H_4_), MK-9(H_6_) and MK-9(H_8_) (9.6%). The major fatty acids (>5%) are iso-C_16:0_, iso-C_15:0_, iso-C_18:0_, C_17:1**ω10**c_, C_16:0_, C_17:0_ and C_15:0_. The characteristic phospholipids are phosphatidylethanolamine, diphosphatidylglycerol, phosphatidylinositol mannoside, phosphatidylinositol, phosphatidylserine and an unidentified phospholipid. The G+C content of the DNA of the type strain is 70.1 mol%.

The type strain, 2603PH03^T^ (=CCTCC AA 2016023^T^ = DSM 45673), was isolated from a mangrove rhizosphere soil sample that was collected in Wenchang, Hainan, China. The GenBank/EMBL/DDJB accession number for the 16S rRNA gene sequence of the type strain 2603PH03^T^ is HQ123438.

## Electronic supplementary material

Below is the link to the electronic supplementary material.

Supplementary material 1 (pdf 816 kb)
