# Supplementary material for: Verrucosispora rhizosphaerae sp. nov., isolated from mangrove rhizosphere soil
Source: Antonie Van Leeuwenhoek. 2017 Sep 22;111(1):125–33. doi: 10.1007/s10482-017-0933-4 (PMC5754459; doi:10.1007/s10482-017-0933-4)

Supplementary Fig. S1. Scanning electron micrograph of substrate mycelium of culture of strain 2603PH03^{T} after growth on ISP 2 agar for 21 days at 28 °C.

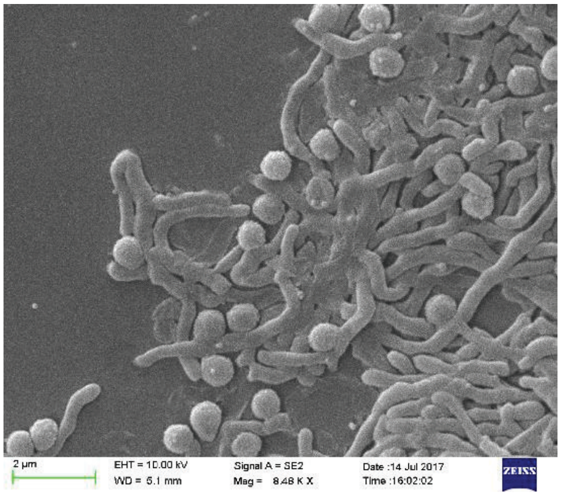

Supplementary Table S1. Cultural characteristics of strain 2603PH03^T and closely related members of the genus *Verrucosispora*.

Media	2603PH03^T	*V. fiedleri* MG-37^T	*V. gifhornensisa* DSM 44337^T	*V. maris* DSM 45365^T
Tryptone-yeast extract agar (ISP1)				
Growth	+++	+++	+++	+++
Color of colony*	Dark orange brown	Orange	Orange	Dark orange brown
Yeast extract-malt extract agar (ISP2)				
Growth	+++	+++	+++	+++
Color of colony*	Dark orange brown	Dark orange brown	Orange	Orange
Oatmeal agar (ISP3)				
Growth	+ +	+++	++	+++
Color of colony*	Orange	Orange	Orange	Orange
Inorganic salt-starch agar (ISP 4)				
Growth	+	+++	++	+++
Color of colony*	Vivid orange yellow	Orange	Vivid orange yellow	Orange
Glycerol-asparagine agar (ISP 5)				
Growth	+	+	++	++
Color of colony*	Vivid orange yellow	Vivid orange yellow	Orange	Orange
Peptone-yeast extract iron agar (ISP 6)				
Growth	+	++	+	+
Color of colony*	Orange	Orange	Vivid orange yellow	Vivid orange yellow
Tyrosine agar (ISP 7)				
Growth	+	++	++	+
Color of colony*	Vivid orange yellow	Orange	Orange	Vivid orange yellow
Tap water agar				
Growth	+	+	+	+
Color of colony*	Orange	Orange	Orange	Orange
GYM agar				
Growth	+++	+++	+++	+++
Color of colony*	Dark orange brown	Dark orange brown	Orange	Orange
Modified Bennett agar				
Growth	+++	++	++	+++
Color of colony*	Dark orange brown	Orange	Orange	Dark orange brown

* The ISCC-NBS color charts were used to determine the designations of colony colors (Kelly 1964).

Supplementary Fig. S2

Two-dimensional TLC of the polar lipid profile of strain 2603PH03^{T}.

Abbreviations: PE, phosphatidylethanolamine; PIMS, phosphatidylinositol mannosides; DPG, diphosphatidylglycerol; PI, phosphatidylinositol; PS, phosphatidylserine; PL, unidentified phospholipids.

a, Sprayed with ninhydrin; b, Sprayed with molybdenum blue; c, Sprayed with phosphomolybdic acid.

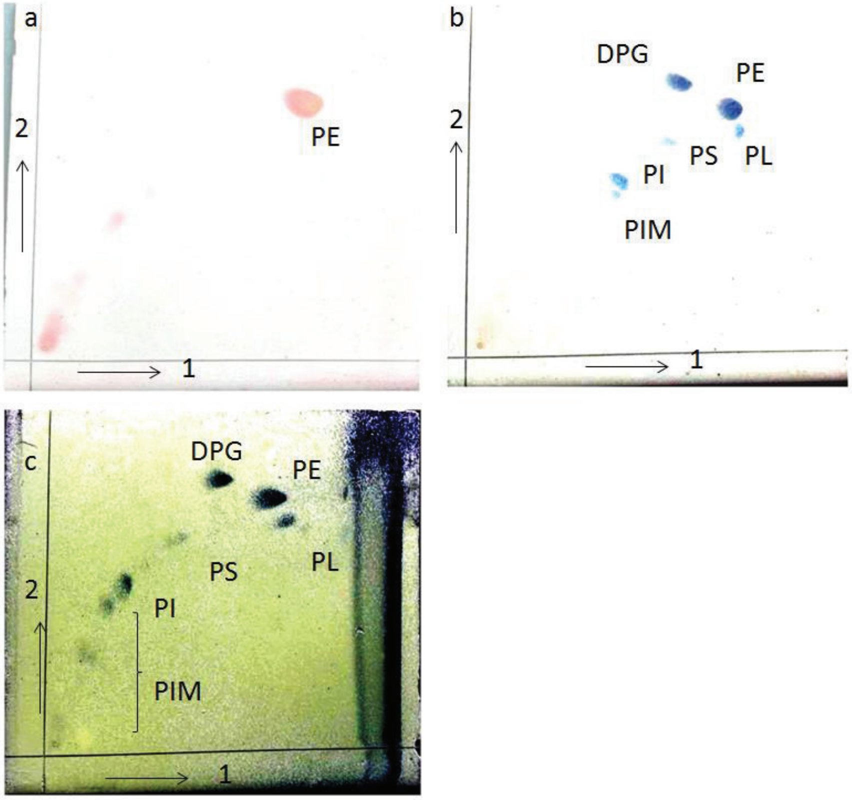

Supplementary Fig. S3. Maximum-likelihood (Felsenstein 1981) (a) and maximum-parsimony (Kluge and Farris 1969) (b) phylogenetic trees, based on almost-complete 16S rRNA gene sequences (1407 nt), showing the relationships between strain 2603PH03^{T} and other members of the genus *Verrucosispora. Salinispora arenicola* CNB-643^{T} was used as an outgroup. Numbers at branch points indicate bootstrap percentages (based on 1000 replicates); only values >50 % are indicated. Bar, 0.002 substitutions per nucleotide position.

(a)

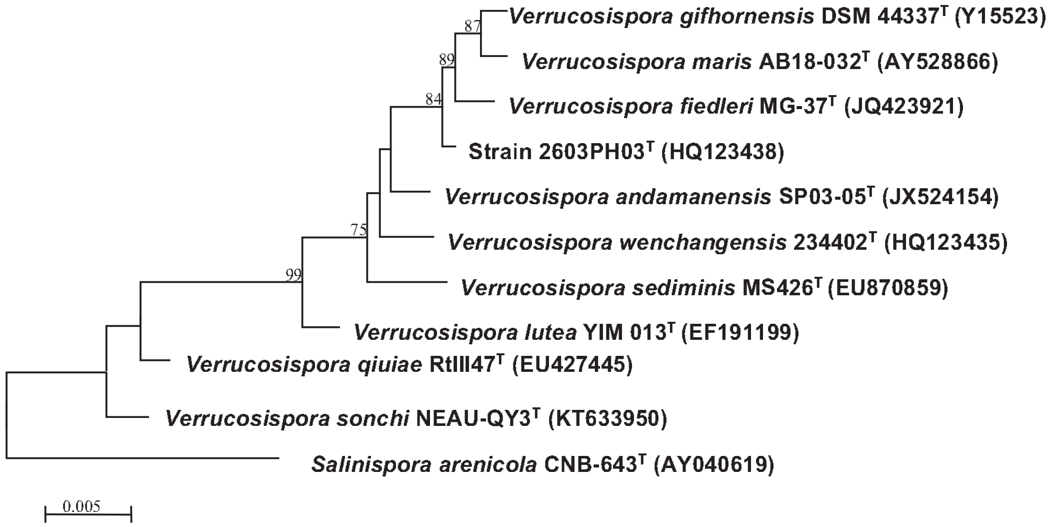

(b)

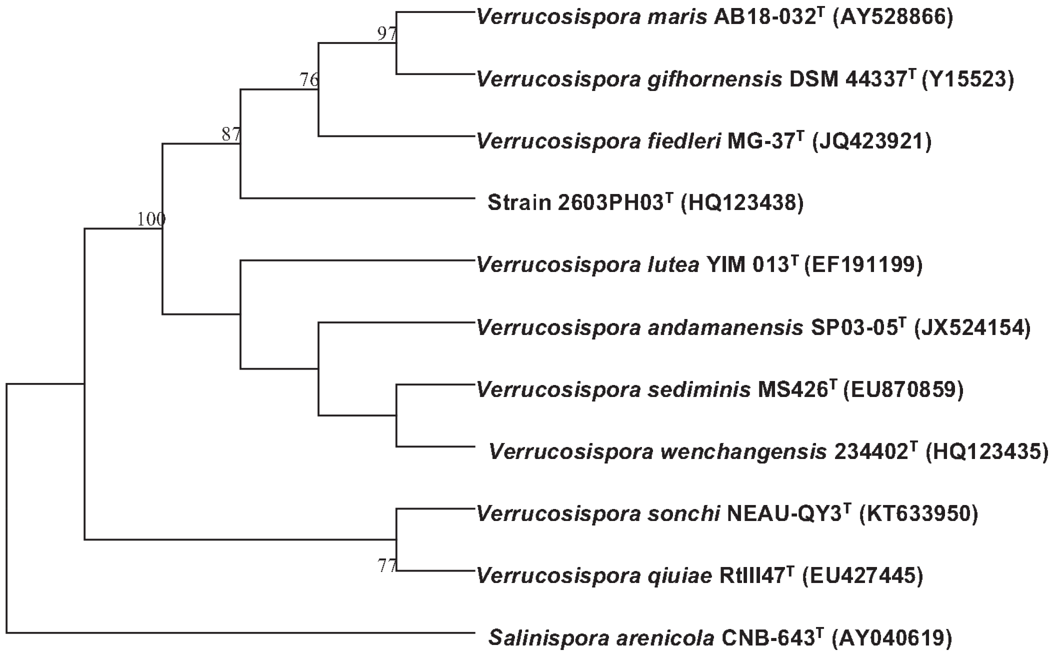

Supplement: Supplementary file 1 — Supplementary material 1 (pdf 816 kb) [file 10482_2017_933_MOESM1_ESM.pdf]
